# Varying patterns of association between cortical large-scale networks and subthalamic nucleus activity in Parkinson’s disease

**DOI:** 10.1038/s41531-026-01372-1

**Published:** 2026-05-02

**Authors:** Oliver Kohl, Chetan Gohil, Matthias Sure, Alfons Schnitzler, Esther Florin

**Affiliations:** 1 Institute of Clinical Neuroscience and Medical Psychology, Medical Faculty, Heinrich-Heine-University, Düsseldorf, Germany; 2 School of Computer Science, University of Sydney, Sydney, NSW Australia; 3 Brain and Mind Centre, University of Sydney, Sydney, NSW Australia; 4 Department of Neurology, Centre for Movement Disorders and Neuromodulation, Medical Faculty, Heinrich-Heine-University, Düsseldorf, Germany

**Keywords:** Neurology, Neuroscience

## Abstract

The rising prevalence of Parkinson’s disease has created an urgent need for brain activity markers guiding diagnosis and treatment strategies. While abnormal basal ganglia activity is known to synchronise with specific cortical regions, the temporal dynamics and cortical network architecture of this coupling remain unclear. To address this, we analysed simultaneous magnetoencephalography and subthalamic nucleus (STN) local field potential recordings from 27 individuals with Parkinson’s disease, both on and off dopaminergic medication. Using a time-delay embedded Hidden Markov Model, we identified dynamic large-scale cortical networks whose occurrences fluctuate over time and exhibit distinct STN-cortical coupling. STN-supplementary motor area (SMA) synchrony increased during activations of the sensorimotor network and the posterior default mode network. The former was associated with 9.5–23 Hz power and beta bursts in the STN, and the latter with 5–16.5 Hz power. Dopaminergic medication preferentially reduced STN beta power in networks lacking enhanced STN-SMA synchrony. These findings suggest that large-scale cortical networks have varying patterns of association with STN activity and may provide temporal windows into subcortical processing. Such network signatures in non-invasive recordings offer promising candidates for markers of subcortical-cortical activity in Parkinson’s disease and may provide targets for treatment strategies, including closed-loop stimulation.

## Introduction

Parkinson’s disease (PD) is the second most common neurodegenerative disorder after Alzheimer’s disease^1^. Its morphological hallmark is the degeneration of dopaminergic neurons in the substantia nigra pars compacta, resulting in increased 13–30 Hz (beta) activity in the subthalamic nucleus (STN)^2–4^, which is underpinned by prolonged occurrences of transient oscillatory events (beta bursts)^5,6^. Dopaminergic medication has been associated with reductions in low-beta power (13–20 Hz), whereas DBS preferentially suppresses high-beta power (21–30 Hz)^7^.

At the network level, STN oscillations synchronise with multiple cortical regions, forming spatially and spectrally distinct subcortical-cortical networks^8–13^. These include an alpha (7–12 Hz) network, involving temporal and brainstem areas^9,10^, and a high-beta (21–30 Hz) network linking the STN with medial frontal areas—including the supplementary motor area (SMA), the premotor, and sensorimotor cortices—via the hyperdirect pathway, a monosynaptic cortico-subthalamic connection^7,8,11,13^. DBS reduces this STN-cortical synchronisation^7,8^, although this reduction does not correlate with motor improvements^8^. Dopaminergic medication has been reported to either reduce SMA-STN high-beta coherence^7^ or have no effect^9–11^.

Synchronisation between the STN and motor cortex increases during STN beta bursts, and in particular, long STN bursts temporally overlap with motor cortical beta bursts^14,15^. Thus, exaggerated beta bursting is hypothesised to reflect periods of heightened synchronisation within the STN-motor cortical network, constraining information processing in the basal ganglia-thalamo-cortical loop^14^. This excessive synchrony is thought to entrain neurons into redundant, rhythmic firing patterns. These patterns, in turn, promote the motor “status quo” by limiting the neural flexibility required for movement initiation, thereby contributing to motor impairments^2,14,16^. Crucially, PD-related alterations in cortical beta bursts occur predominantly within specific cortical networks^17^, suggesting that examining the association between dynamic cortical networks and STN beta bursts may further refine our understanding of pathological synchronisation within the basal ganglia-thalamo-cortical loop.

The extent to which periods of elevated STN-cortical connectivity can be detected through non-invasive recordings of cortical oscillatory activity remains an open question. Therefore, we investigate whether large-scale cortical networks derived from non-invasive MEG recordings exhibit distinct patterns of STN-cortical connectivity. We specifically hypothesise that sensorimotor network activations are associated with increased STN-SMA synchronisation via the hyperdirect pathway^11^, consistent with previous reports for STN beta bursts^14^. Building on the idea that dynamic cortical networks provide temporal windows into distinct periods of STN activity, we expect sensorimotor network visits to coincide with STN beta bursts^14,15^ and elevated STN beta power, highlighting the importance of the sensorimotor network. Finally, we explore whether other dynamic cortical networks similarly pinpoint distinct STN activity states, and whether dopaminergic medication affects STN beta activity associated with specific cortical networks, a question with direct relevance to understanding how pharmacological treatment normalises pathological STN hypersynchronisation. Identifying reliable cortical signatures of STN-cortical connectivity could inform the development of adaptive neuromodulation strategies^15,18^ and provide non-invasive electrophysiological markers of PD.

## Results

To investigate the relationship between large-scale cortical networks and subcortical STN activity, we analysed simultaneously recorded MEG and STN-LFP data of 27 individuals with Parkinson’s Disease (see Table 1 for details) in the dopaminergic medication on and off condition. STN-LFP recordings were conducted through externalised leads 1 to 3 days after the surgery, while an eyes-open resting-state MEG scan was performed.

**Table 1:** Descriptive statistics of included participants

Demographics	Düsseldorf dataset		Rassoulou et al.
	HC	PD	PD
*N*	25	25	17
Sex (female/male)	8/17	7/18	6/12
Age (years) (mean, range)	63.36 (54-72)	59.32 (41–72)	63.35 (48–76)
Years since diagnosis (years) (mean, range)	N/A	7 (1–15)^*^	10 (1–21)
MDS-UPDRS III motor score On (mean, range)	N/A	20.17 (8-38)^*^	21.94 (9–36)
MDS-UPDRS III motor score Off (mean, range)	N/A	30 (14–16)^*^	37.17 (24–55)
Administered Levodopa dose (mg)^**^ (mean, range)	N/A	177 (100–300)	N/A

*N* number of participants, *MDS-UPDRS* revised unified Parkinson disease rating scale, *N/A* not available.

^*^Values were computed across all participants for whom score data were available.

^**^Dose of standardised fast-acting levodopa administered to induce Medication-On state.

### STN beta power is modulated by dopaminergic medication

**Fig. 1:**
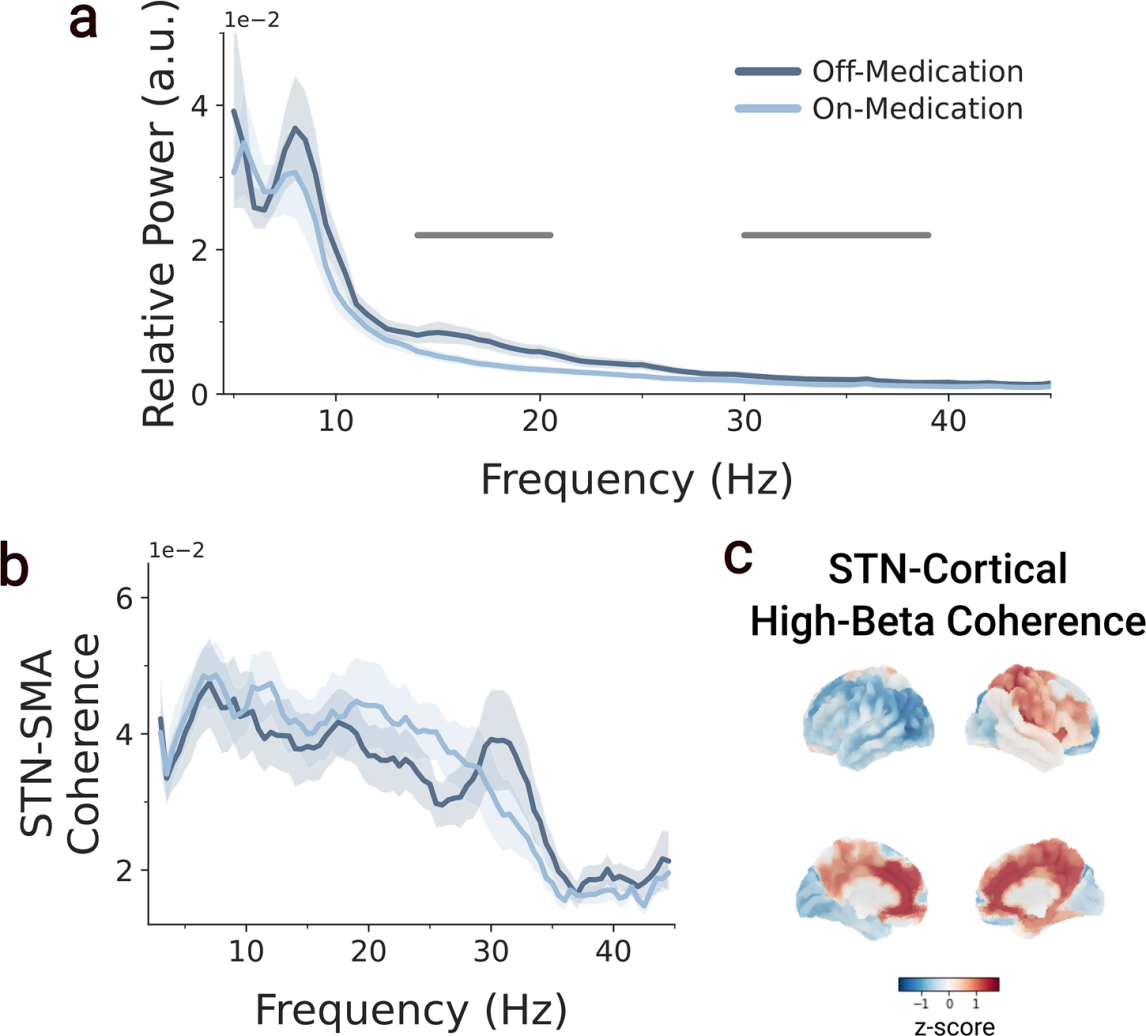
Effect of dopaminergic medication on time-average STN spectral power and STN-SMA coherence. **a** Average power spectrum of the PD group in the medication off (dark blue) and medication on (light blue) conditions. Two clusters with significantly increased power in the beta (peak frequency = 16-Hz; mean *t*(23) = −2.59, *p* = 0.019) and gamma range (peak frequency = 35-Hz; mean *t*(23) = −2.3, *p* = 0.011), obtained from the cluster-based permutation test, are marked with a bar. **b** Average coherence spectrum of PD group in medication off (dark blue) and medication on (light blue) conditions. Cluster-based permutation tests did not yield clusters with significant group differences. **c** Average STN-cortical high-beta coherence across the whole brain in the medication Off State. Z-scored coherence values were flipped so that the ipsilateral coherence values of both hemispheres are depicted on the right hemisphere and the contralateral coherence on the left hemisphere.

First, we investigate time-averaged oscillatory power and coherence without considering potential temporal variations of these measures to replicate previous work^2–4,8^. Oscillatory power averaged across the best clinical contact of each hemisphere was calculated. General linear models (GLMs) and cluster-based permutation tests identified a 14 to 21.5 Hz cluster (peak frequency = 16-Hz; mean *t*(23) = −2.59, *p* = 0.019) and a 30 to 40-Hz cluster with significantly reduced power (peak frequency = 35-Hz; mean *t*(23) = −2.3, *p* = 0.011) in the medication on condition (Fig. 1a). In contrast, the medication status of the PD volunteers did not significantly alter STN-SMA coherence (Fig. 1b). Repeated measures correlations between STN beta power and Bradykinesia/rigidity scores in the medication-off condition demonstrated a non-significant trend towards a positive association (*r*(23) = 0.38, *p* = 0.06), similar to previous reports^19^.

### Dynamic large-scale cortical networks

**Fig. 2:**
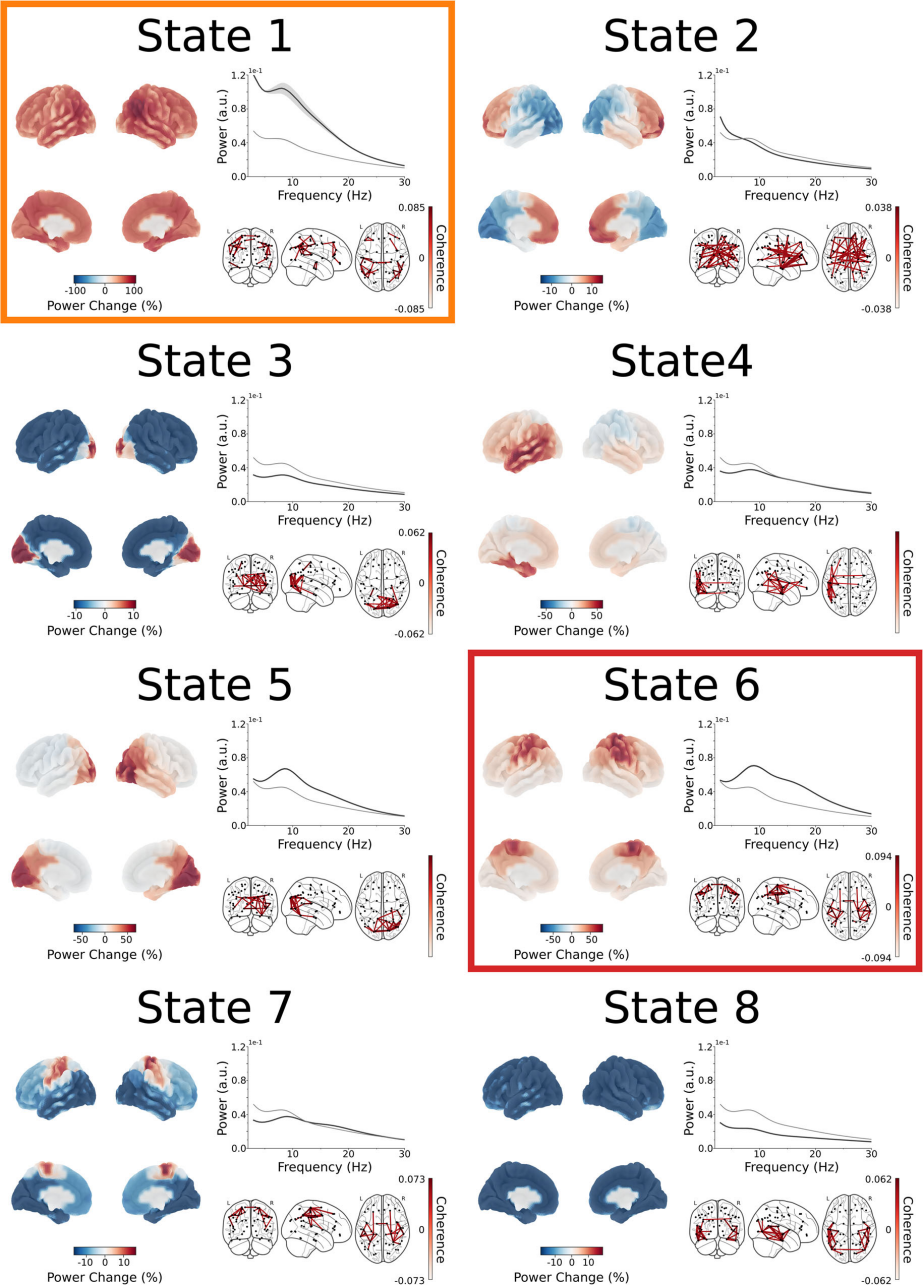
Overview of dynamic large-scale cortical networks inferred using the TDE-HMM. Each state is presented in three panels, showing the average across scans from all participants in both the medication-off and -on conditions. Left panel: Deviations in 3 to 30-Hz power from the time-averaged power (calculated across all states) are projected onto the cortical surface. Top right panel: The state-specific motor cortical power spectrum (black) is displayed alongside the time-averaged power spectrum across all states (grey). Bottom right panel: Coherence networks in the 2 to 30-Hz range, thresholded at the 98th percentile, to highlight the most prominent functional connections. States of particular relevance to this study are marked with coloured boxes: State 1 (orange), representing the posterior default mode network, and State 6 (red), corresponding to a sensorimotor network.

A time-delay embedded hidden Markov model (TDE-HMM) was applied to the parcellated MEG data to extract temporally and spectrally resolved, large-scale cortical networks. Critically, network identification was performed by fitting a set of canonical network descriptions previously learned from a normative dataset^20^ to the MEG data, enabling robust network inference despite the increased noise associated with externalised leads and implanted DBS electrodes. This analysis yields moment-by-moment estimates of network occurrence, along with network-specific spectral characteristics computed from time periods in which each network is most active. The resulting network states (Fig. 2) closely resemble networks reported in previous studies using TDE-HMMs to characterise dynamic brain networks^17,21–23^. Fractional occupancy values, quantifying the proportion of time spent in each network state, ranged from 1 to 55% across participants and states. This distribution indicates that all identified states were visited by each participant, and that no individual’s data were dominated by a single state. State 6 likely reflects the sensorimotor network, as it was characterised by marked increases in wideband (2–30 Hz) power localised to sensorimotor regions. The associated power spectrum displayed a prominent beta-band peak alongside an alpha peak, consistent with known spectral features of sensorimotor activity^24^. State 1 exhibited an even greater increase in sensorimotor wideband power; however, this increase was not confined to sensorimotor areas but was broadly distributed across the cortex. This spatially widespread activation state has previously been related to the posterior part of the default mode network^23,25,26^.

Further, comparisons between fractional occupancy of the sensorimotor network in the PD group in the off-medication condition and a healthy control group matched in age and sex, yielded a significant reduction in the PD group (*t*(47) = 4.48, *p* < 0.001) that likely underpinned motor cortical beta power decreases in the PD group (*t*(47) = 6.78, *p* < 0.001) (Supplementary Fig. 1), replicating previous research^17^.

Based on these spatial and spectral characteristics, we refer to State 6 as the sensorimotor network and State 1 as a posterior default mode network (pDMN).

### High-beta coherence is increased during sensorimotor network and posterior default mode network visits

We next examined whether large-scale cortical networks exhibit distinct STN-SMA coherence patterns. Network-specific coherence was computed using a multi-taper approach, weighting MEG–LFP recordings with time-resolved network-occurrence probability time courses before coherence calculation. State-specific coherence spectra were compared to the time-averaged STN-SMA spectrum using GLMs and cluster-based permutation tests. Cluster-level *p* values were Bonferroni corrected to account for multiple comparisons across the eight networks.

**Fig. 3:**
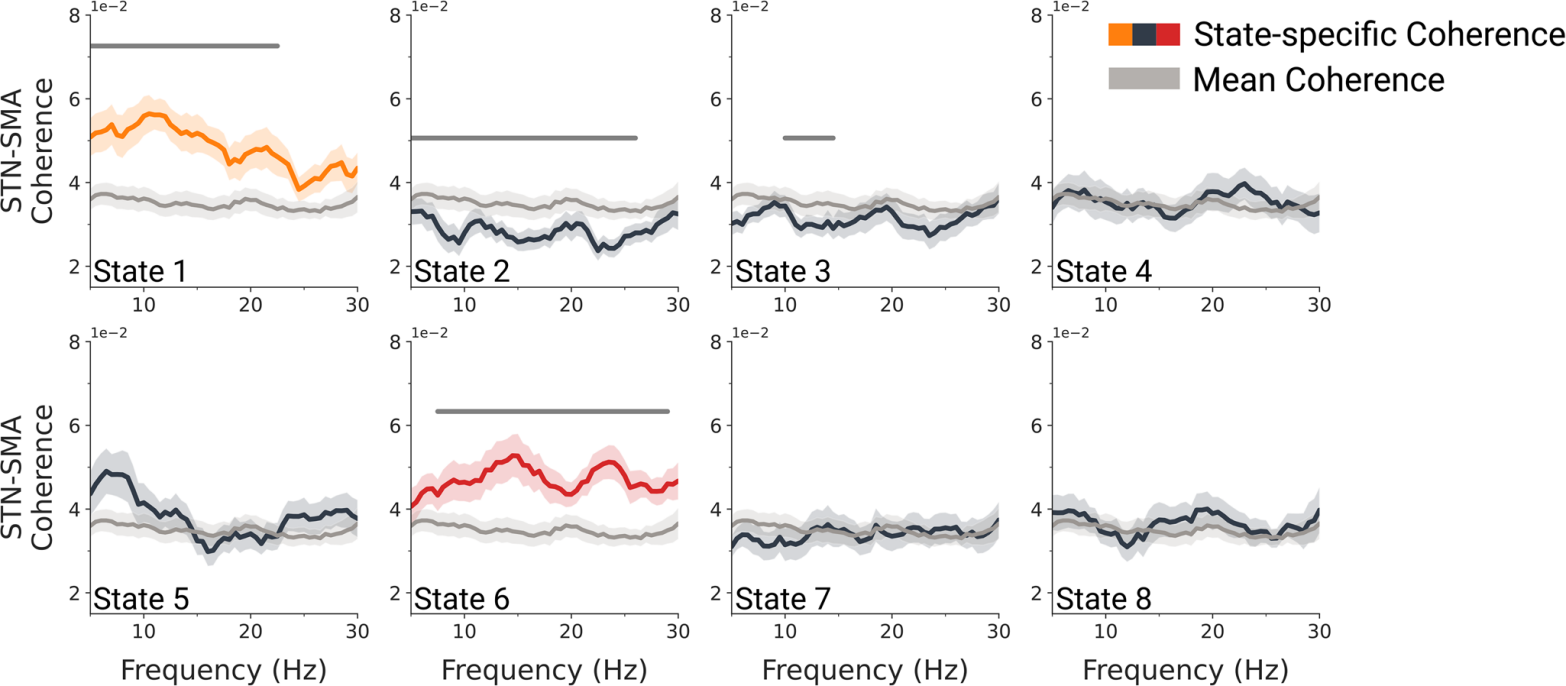
STN-SMA coherence varies across dynamic cortical networks. State-specific STN-SMA coherence (dark grey, dark blue, dark orange) is plotted alongside the time-averaged STN-SMA coherence (averaged across all networks). Within-participant GLMs combined with cluster-based permutation testing revealed that only the posterior default mode network (State 1; orange; peak frequency = 12 Hz; mean *t*(23) = 4.43, *p* < 0.001) and the sensorimotor network (State 6; red; peak frequency = 22 Hz; mean *t*(23) = 4.1, *p* < 0.001) had clusters with significantly increased coherence. In contrast, State 2 (peak frequency = 24 Hz; mean *t*(23) = −3.58, *p* < 0.001) and State 3 (peak frequency = 13.5 Hz; mean *t*(23) = −2.85, *p* = 0.003) exhibited significantly decreased STN-SMA coherence. Grey bars indicate clusters with *p* values below the Bonferroni-corrected threshold for 8 state comparisons (*p* < 0.00625).

This analysis identified two networks with increased STN-SMA coherence relative to the time-averaged baseline (coherence averaged across all network states). Within-subject GLMs and cluster-based permutation testing revealed a significant cluster spanning 5 to 23.5 Hz with increased STN-SMA coherence during pDMN visits (peak frequency = 12 Hz; mean *t*(23) = 4.43, *p* < 0.001). Similarly, STN-SMA coherence during sensorimotor network visits was significantly elevated in the 7.5 to 30-Hz range (peak frequency = 22 Hz; mean *t*(23) = 4.1, *p* < 0.001) (Fig. 3). To ensure that observed fluctuations in STN-SMA coherence were not solely driven by variations in SMA power across state visits, these effects were further confirmed by a control analysis using absolute imaginary coherence, yielding comparable results when comparing state-specific coherence to time-averaged coherence (Supplementary Fig. 2). Furthermore, we observed decreased STN-SMA coherence during activation of two other networks: a frontal network (State 2) and a visual network (State 3). Specifically, the frontal network showed reduced coherence in the 5 to 27 Hz range (peak frequency = 24 Hz; mean *t*(23) = –3.58, *p* < 0.001), while the visual network exhibited a reduction in the 10 to 15.5 Hz band (peak frequency = 13.5 Hz; mean *t*(23) = –2.85, *p* = 0.003). Overall, these findings indicate that STN-SMA coherence dynamically varies across distinct cortical large-scale networks, solely identified from non-invasive MEG recordings.

### Cortical networks dissect distinct STN spectral profiles

**Fig. 4:**
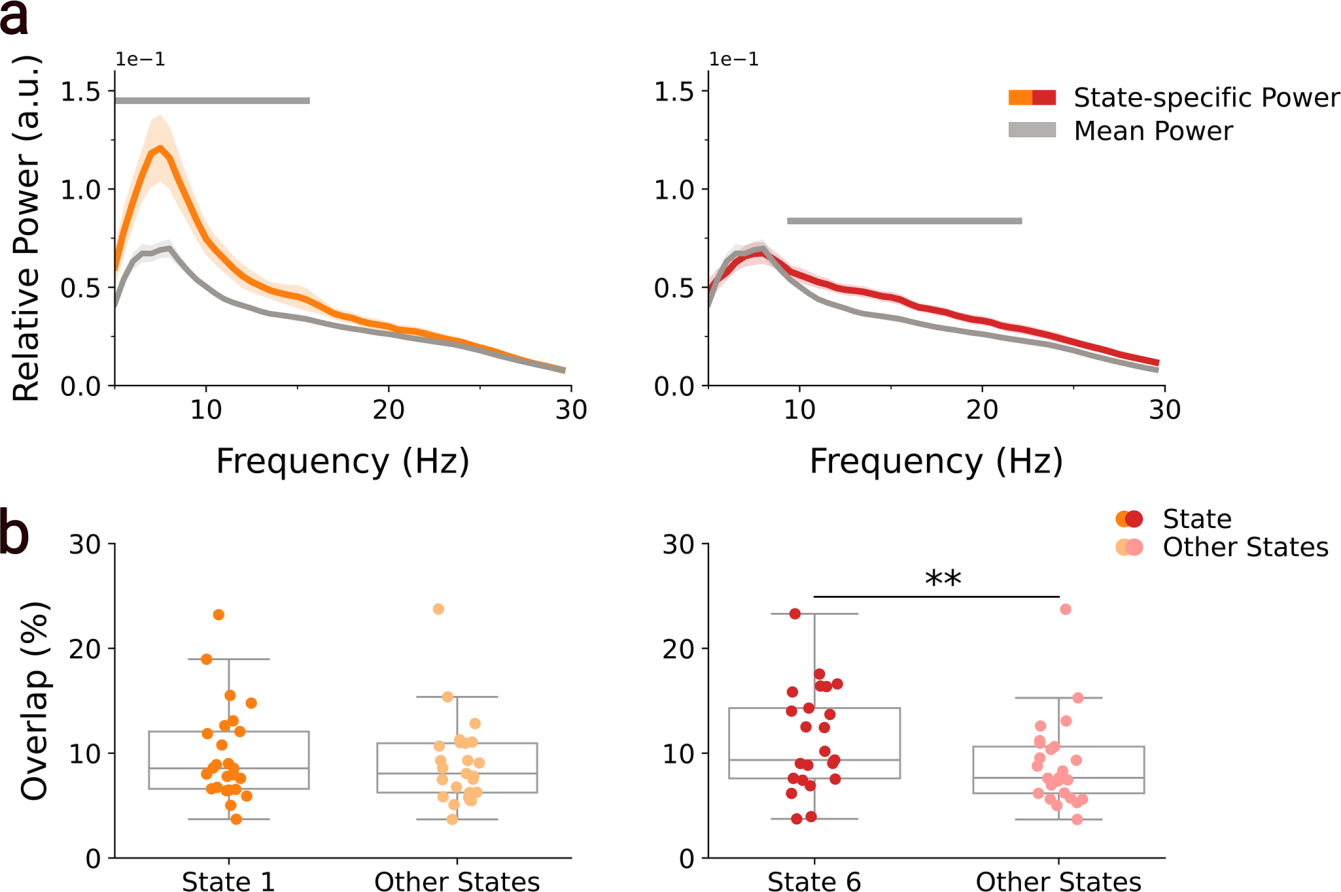
The posterior default mode and sensorimotor networks segment distinct STN spectral profiles. **a** State-specific STN power spectra for the posterior default mode (State 1; orange) and the sensorimotor network (State 6; red) are shown alongside the time-averaged STN power spectrum across all states (grey). Cluster-based permutation testing on within-participant GLMs revealed significantly increased STN power during visits to the posterior default mode network (5 to 16.5 Hz; peak = 6.5 Hz; mean *t*(24) = 2.91, *P* < 0.001) and the sensorimotor network (9.5 to 23 Hz; peak = 14 Hz; mean *t*(24) = 3.41, *P* = 0.001). Grey bars indicate clusters surviving Bonferroni correction for eight state comparisons (*P* < 0.00625). **b** Temporal overlap between STN beta bursts and cortical network occurrences. Beta bursts significantly overlapped with the sensorimotor network (dark red; *t*(24) = 3.6, *P* = 0.001), but not with the posterior default mode network (dark orange; *t*(24) = 1.93, *P* = 0.121), based on within-participant GLMs. Asterisks denote statistical significance: **p < 0.01; *p < 0.05.

After observing that different large-scale cortical networks exhibit distinct patterns of STN-SMA synchrony, we next investigated whether the occurrence of these networks, identified solely from non-invasive MEG data, dissected distinct STN activity profiles. To do this, we computed STN oscillatory power during periods when each network was active. The resulting state-specific power spectra were contrasted against the time-averaged STN power spectrum (averaged across all networks) using GLMs and cluster-based permutation testing to identify frequency clusters with significant differences. This procedure was repeated across all eight networks, and the significance of the resulting cluster *p* values was assessed with Bonferroni-corrected significance thresholds. Critically, the two networks that exhibited increased STN-SMA coherence also had significant increases in STN power. During visits to the pDMN, STN power was significantly elevated in the 5 to 16.5 Hz range (peak frequency = 6.5 Hz; mean *t*(24) = 2.91, *p* < 0.001). Similarly, during activations of the sensorimotor network, we observed increased STN power in the 9.5 to 23 Hz range (peak frequency = 14 Hz; mean *t*(24) = 3.41, *p* = 0.001) (Fig. 4a). No other networks showed significant increases in STN power (Supplementary Fig. 3). To further assess whether these network-specific increases in STN power reflect increased co-occurrence with STN beta bursts, we examined the temporal overlap between STN beta bursts and occurrences of the pDMN and sensorimotor network. Specifically, we compared the overlap of STN beta bursts with these two networks against beta bursts occurring during all other network states. This analysis revealed that beta bursts significantly overlapped with the sensorimotor network (*t*(24) = 3.6, *p* = 0.001), but not with the pDMN (*t*(24) = 1.93, *p* = 0.121) (Fig. 4b).

#### Medication-related STN beta power reductions are most pronounced during cortical network occurrences that do not show increased STN-cortical coherence

**Fig. 5:**
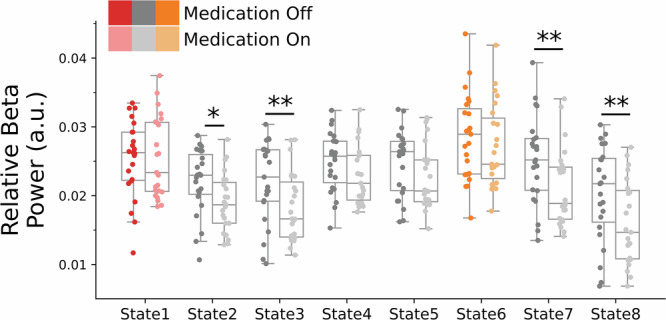
Effects of dopaminergic medication on STN beta power are most pronounced during cortical networks not associated with increased STN-SMA coherence. State-specific average STN power in the 13 to 30-Hz range was contrasted between the medication-off and -on conditions using within-participant GLMs and maximum t-statistic permutation testing to correct for multiple comparisons across states. While STN beta power was qualitatively reduced by medication across all network states, significant reductions were observed only during visits to State 2 (*t*(24) = 3.37, *p* = 0.012), State 3 (*t*(24) = 3.82, *p* = 0.003), State 7 (*t*(24) = 4.06, *p* = 0.003), and State 8 (*t*(24) = 3.69, *p* = 0.005). Asterisks denote statistical significance: **p < 0.01; *p < 0.05.

We found that time-averaged STN beta power (13–30 Hz) was reduced following the intake of dopaminergic medication. Having shown that visits to distinct large-scale cortical networks correspond to different STN oscillatory profiles, we next explored whether the effect of medication on STN beta power is more pronounced during specific cortical networks. To assess this, we computed state-specific STN beta power for each network in both the medication-on and medication-off conditions. Qualitative observations indicate that STN beta power was consistently higher in the off-medication condition across all networks. To formally test for state-dependent effects, we conducted within-participant contrasts using GLMs and maximum *t*-statistic permutation testing, pooling across states to correct for multiple comparisons. This analysis revealed significant reductions in STN beta power during visits to State 2 (*t*(24) = 3.37, *p* = 0.012), State 3 (*t*(24) = 3.69, *p* = 0.005), State 7 (*t*(24) = 4.06, *p* = 0.003), and State 8 (*t*(24) = 3.59, *p* = 0.008) in the medication-on condition (Fig. 5). Before this analysis, STN beta power values from two participants were excluded as outliers based on a generalised extreme studentized deviate (GESD) test. Importantly, repeating the analysis with these outliers included yielded a similar pattern: significant beta power reductions were observed in State 3 (*t*(24) = 3.82, *p* = 0.003), State 7 (*t*(24) = 3.22, *p* = 0.016), and State 8 (*t*(24) = 3.69, *p* = 0.005). Interestingly, none of the states previously associated with elevated STN-SMA coherence and increased STN beta power were significantly affected by medication, suggesting that dopaminergic medication effects may be particularly pronounced during network states characterised by lower STN-SMA coherence.

### Robustness analysis

TDE-HMM analyses require a priori specification of the number of inferred states. As standard model selection metrics often fail to identify a clear optimal number of states^20^, we repeated all analyses using TDE-HMMs inferring 10 and 12 States. The results demonstrate that reported findings are robust to the choice of state number and not restricted to models with 8 states (Supplementary Figs. 4 and 5).

### Replication analysis

**Fig. 6:**
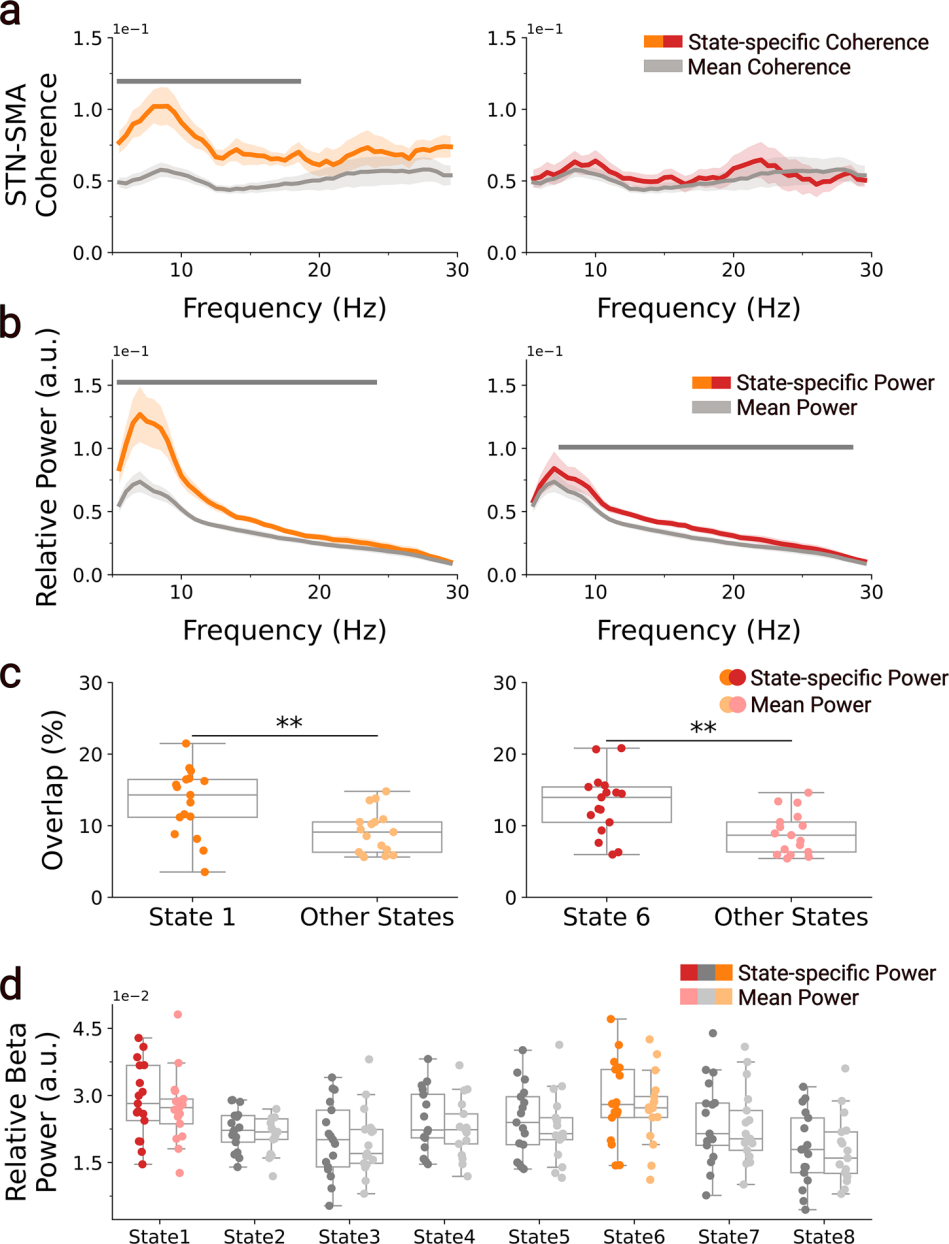
Replication of cortical network-specific modulation of STN-SMA coherence and STN oscillatory activity in an independent LFP-MEG dataset^27^. **a** State-specific STN-SMA coherence (dark orange, dark red) is shown alongside the time-averaged coherence across all states (grey). Within-participant GLMs, combined with cluster-based permutation testing, revealed significantly increased STN-SMA coherence during visits to the pDMN (State 1; orange; 5.5–18.5 Hz; peak = 12 Hz; mean *t*(16) = 4.16, *p* < 0.001), whereas no significant clusters were found for the sensorimotor network. **b** State-specific STN power spectra for the pDMN (orange) and sensorimotor network (red) are shown alongside the time-averaged STN power spectrum (grey). Visits to the pDMN were associated with significantly increased power in the 5.5 to 25.5-Hz range (peak = 14.5 Hz; mean *t*(16) = 4.45, *p* < 0.001), and the sensorimotor network in the 7.5 to 29.5-Hz range (peak = 18 Hz; mean *t*(16) = 5.33, *p* < 0.001). **c** Temporal overlap between STN beta bursts and cortical network occurrences revealed significant co-occurrence with the sensorimotor (dark red; *t*(16) = 6.70, *p* < 0.001) and pDMN (dark orange; *t*(16) = 5.02, *p* < 0.001) networks, based on within-participant GLMs. **d** State-specific STN beta power (13–30 Hz) was compared between the medication-off and -on conditions using within-participant GLMs and maximum *t*-statistic permutation testing. No significant medication effects were observed in any network state (all *t*(16) ≤ 2.33, *P* = 0.138). Grey bars indicate clusters with *p* < 0.00625 (Bonferroni-corrected threshold for eight states). Asterisks denote statistical significance: **p < 0.01; *p < 0.05.

Having demonstrated that dynamic large-scale cortical networks exhibit distinct patterns of STN-SMA coherence and STN oscillatory activity, we aimed to assess the generalisability of these findings using an independent dataset. To this end, we preprocessed a publicly available dataset^27^ containing simultaneous MEG–LFP recordings of 20 individuals with PD and repeated all analyses. An overview, with state descriptions of the extracted dynamic large-scale cortical networks, can be found in Supplementary Fig. 6. GLMs comparing state-specific STN-SMA coherence against time-averaged coherence, combined with cluster-based permutation testing (Fig. 6a), revealed a significant increase in 5.5 to 18.5-Hz coherence during visits to the pDMN (peak frequency = 12 Hz; mean *t*(16) = 4.16, *p* < 0.001). No significant cluster was found in the STN-SMA coherence during sensorimotor network activations, although repeating the analyses for the premotor cortex, located right next to the SMA, yielded a 15.5 to 21.5-Hz cluster with significantly increased STN-premotor cortex coherence (peak frequency = 19 Hz; mean *t*(16) = 3.64, *p* = 0.002) (Supplementary Fig. 7). Similarly, comparisons between state-specific and time-averaged STN power spectra replicated significant increases in STN oscillatory power, albeit with broader spectral profiles (Fig. 6b). Specifically, we observed a 5.5 to 25-Hz cluster with increased power during pDMN visits (peak frequency = 14.5 Hz; mean *t*(16) = 4.45, *p* < 0.001), and a 7.5 to 29.5-Hz cluster during sensorimotor network visits (peak frequency = 18 Hz; mean *t*(16) = 5.33, *p* < 0.001). Consistent with these broadband increases in STN power, STN beta bursts significantly overlapped with both pDMN (*t*(16) = 5.02, *p* < 0.001) and sensorimotor network (*t*(16) = 6.70, *p* < 0.001) occurrences (Fig. 6c). GLMs combined with cluster-based permutations comparing time-averaged STN power spectra between medication-on and -off conditions did not yield significant differences. Likewise, medication did not significantly modulate state-specific STN beta power for any of the cortical networks (all *t*(16) ≤ 2.33, *p* = 0.138) (Fig. 6d). In summary, we successfully replicated the core findings, cortical network-specific modulation of STN-SMA coherence and STN oscillatory activity, in an independent dataset. The only exception was the absence of significant medication effects. Notably, spectral effects in this dataset were less frequency-specific than those observed in our primary dataset.

## Discussion

In the present study, we examined whether large-scale cortical networks extracted from non-invasive MEG recordings exhibit distinct patterns of STN-cortical connectivity and can index variations in STN activity. We found that specific networks (sensorimotor and pDMN) demonstrate enhanced STN-SMA coherence. During visits to these networks, oscillatory power in the STN exhibited distinct spectral signatures: the sensorimotor network was associated with increased power in the 9.5 to 23-Hz range and co-occurred with STN beta bursts, whereas power in the pDMN was increased in the 5 to 16.5-Hz range. Similar patterns were replicated in an independent dataset, albeit with slightly coarser spectral specificity. Medication-related reductions in STN beta power, evident in the time-averaged spectrum, were most pronounced during network occurrences not associated with increased STN-cortical coherence, though this pattern was observed only in the main dataset and did not replicate. Together, these results indicate that large-scale cortical networks provide temporally resolved windows into distinct STN activity states, offering a potential framework for probing subcortical-cortical interactions relevant to PD and for informing future diagnostic and therapeutic strategies.

We demonstrated that periods with increased STN-motor cortical coherence can be detected non-invasively from cortical network dynamics alone, extending previous research that identified elevated STN-motor cortical coupling during invasively recorded STN beta bursts^14^. In particular, the sensorimotor network and pDMN demonstrated increases in STN-SMA coupling: During visits to the sensorimotor network, STN-SMA low- and high-beta coherence was increased. High-beta coherence has been previously linked to hyperdirect pathway activity^7,8,11^, while low-beta coherence was linked to motor outputs and hypothesised to reflect STN-cortical communication via the indirect pathway^7,13^. Therefore, it is likely that both hyperdirect and in- direct pathway processes are embedded in periods in which the sensorimotor network is active. The pDMN, associated with whole-brain increases in wideband power, demonstrated increases in theta-to-alpha coherence between the STN and SMA. In previous studies, alpha coherence between the STN and temporal and cerebellar regions has been reported^9,10,12^. These studies used dynamic imaging of coherent sources (DICS) beamforming and mass-univariate testing with multiple comparison-adjusted significance thresholds, an approach that may be less sensitive to weaker synchronisation patterns. Therefore, our focus on the SMA may have allowed us to observe weaker STN-SMA connections, which are part of this widespread theta-to-alpha network. Given that the pDMN has been hypothesised to support attentional^9^ and sensory processes^10^, and that DMN connectivity has been linked to cognitive performance in PD^28^, increases in STN-cortical synchronisation during pDMN visits may be involved in non-motor symptoms. However, as we did not assess non-motor symptoms, this hypothesis should be interpreted with caution. All in all, our findings demonstrate that information about cortical large-scale networks, solely extracted from non-invasive electrophysiological recordings, allows us to dissect periods of heightened STN-cortical coupling, expanding our knowledge of the spatiotemporal dynamics of previously reported STN-cortical networks.

Crucially, we not only observed that cortical large-scale networks identify periods with increased STN-cortical coupling but also marked periods with distinct STN oscillatory activity. Occurrences of the pDMN and sensorimotor network were associated with periods of increased STN oscillatory activity, albeit with distinct spectral profiles. For the sensorimotor network, beta power was predominantly elevated, whereas for the pDMN, low-frequency power increased. This dissociation demonstrates that non-invasively recorded dynamic cortical networks can dissect varying STN activity states, highlighting the utility of cortical networks as a proxy for STN activity. It remains unclear whether observed associations generalise to people who did not have DBS surgery; however, it is tempting to speculate that cortical network occurrences may offer a non-invasive window into STN functioning in wider PD populations.

Dopaminergic medication tended to reduce STN beta power across all cortical networks, but this effect was most pronounced during visits to cortical networks not associated with increased STN-cortical connectivity. Prior work has reported that dopaminergic medication significantly reduces STN beta activity^2–4^. Our findings refine these results by linking STN beta power reductions to time periods not associated with increased basal-ganglia-thalamo-cortical loop connectivity. This preferential suppression of STN beta power during low-connectivity states could reflect a mechanism by which dopaminergic medication enhances network flexibility through reducing pathological synchronisation^14,16^, particularly in states in which no restriction of information processing capacity throughout the basal ganglia-thalamo-cortical loop is required. This hypothesis should be treated with caution, as we were unable to replicate these medication effects in the replication dataset. The lack of network-specific STN beta power reductions due to dopaminergic medication in the replication dataset is not surprising, given that no medication effects were observed in the time-averaged STN-beta power. Repeating this analysis in an independent dataset in which well-established medication effects on STN beta power can be observed in time-averaged STN power spectra would provide a more definitive test of whether medication preferentially suppresses STN beta hypersynchronisation during periods of reduced basal ganglia-thalamo-cortical loop engagement.

Unlike previous studies on STN-cortical associations^8–11,14,15^, we compared motor cortical activity between age-matched HCs and individuals with PD. Consistent with reports in early-stage PD patients (~2.68 years after their initial PD diagnosis) without implanted DBS^17^, we found reduced sensorimotor network occurrence probability in our PD sample (~7 years after PD diagnosis), indicating that this alteration persists across different disease progressions. Because these alterations can be detected non-invasively, they hold promise as clinically useful biomarkers applicable across the course of the disease. However, their clinical value will depend on future studies: longitudinal work is needed to determine whether these network changes track disease progression, and studies in de novo or at-risk individuals must establish whether they are present in prodromal stages. If confirmed, sensorimotor network dynamics could support disease monitoring and possibly early diagnosis. In this context, our findings represent a first step toward identifying clinically relevant network-based markers for PD. (For a discussion of the sensorimotor network findings in the context of the basal ganglia-thalamo-cortical loop, see Supplementary Information 1).

Our results indicate that dynamic cortical network activity can provide temporal windows to distinct STN activity states, a finding that supports the generation of new hypotheses for developing closed-loop DBS protocols. Large-scale cortical network occurrences may help identify time windows in which adaptive stimulation is most likely to modulate distinct STN-cortical connectivity or STN-oscillatory states. For example, stimulating during sensorimotor network occurrences could target beta synchronisation in the basal ganglia-thalamo-cortical loop, whereas stimulating during pDMN occurrences could modulate alpha STN-cortical coherence. Furthermore, our finding that dopaminergic medication effects are strongest during states without elevated STN-cortical beta coherence suggests that DBS could emulate these effects by preferentially stimulating during such periods. This may reduce STN hypersynchronisation when beta coupling is not functionally relevant. To test these hypotheses about network-specific stimulation targets, our MEG findings must be mapped onto spatially constrained ECoG grids, used in closed-loop DBS research. Although large-scale cortical networks cannot be directly inferred from ECoG recordings, the spatial distribution of activations across ECoG contacts may help to distinguish periods associated with different network activations. Characteristic changes in motor cortical oscillations—such as simultaneously increased alpha and beta power during sensorimotor network visits, selectively increased theta-to-alpha power during pDMN visits, and differences in beta burst waveform shapes^29^—could further serve as markers of specific network occurrences. However, since large-scale cortical network activity is likely also sensitive to widespread cortical oscillatory changes in Parkinson’s disease^30–38^, these targets may need further refinement to ensure specificity and clinical utility. Nevertheless, our findings highlight the potential of using cortical oscillatory signals to identify time windows of clinically relevant STN activity.

In sum, the present study demonstrated that large-scale cortical networks extracted from non-invasive MEG recordings exhibit distinct patterns of STN-cortical connectivity and can index variations in STN activity states. These results indicate that large-scale cortical networks provide temporally resolved windows into distinct STN activity states, offering a potential framework for probing subcortical-cortical interactions relevant to PD and for informing future diagnostic and therapeutic strategies.

## Methods

### Subjects and experimental design

A previously acquired dataset^39–41^ was analysed. Data from a total of 27 patients who underwent bilateral implantation of DBS electrodes into the dorsal STN at the Department of Functional Neurosurgery and Stereotaxy in Düsseldorf were included in this study. These data were complemented by recordings from 25 age and sex-matched healthy controls. Written and informed consent was obtained prior to participation. The study was approved by the local Ethics Committee at the University of Düsseldorf (5608R) and followed the Declaration of Helsinki. A more detailed description of sample demographics and clinical variables can be found in Table 1.

### Data acquisition

MEG recordings were acquired from all participants ~1 day after DBS electrode implantation (range: 1–4 days post-surgery). During data acquisition, STN electrodes were externalised via the St. Jude Medical Directional Extension 6373 (Abbott Laboratories, Lake Bluff, IL, USA) and connected to the EEG amplifier of a 306-channel whole-head MEG system (Elekta Neuromag, Helsinki, Finland), enabling simultaneous recording of LFPs and MEG signals. No electrical stimulation was applied during the analysed recording sessions.

Resting-state data were collected while participants maintained visual fixation on a central black cross. Recordings were conducted in three 10-min blocks for each medication state (OFF and ON dopaminergic medication), resulting in a total acquisition time of 60 min per participant. For the OFF-medication condition, participants withheld dopaminergic medication for at least 12 h. The ON-medication state was induced by administering a standardised fast-acting soluble levodopa dose equivalent to 1.5 times the patient’s regular morning dose. Subsequent scans and assessments were performed at least 30 min after levodopa intake. Motor symptom severity was assessed before each of these MEG sessions using the revised version of the Unified Parkinson’s Disease Rating Scale Part III (MDS-UPDRS III).

### MEG preprocessing

Initially, the raw MEG data were processed using MNE-Python’s Maxwell filtering pipeline. This included automatic detection of bad channels and application of temporal signal-space separation (tSSS) to suppress magnetic signals originating from sources outside the head^42,43^. The automatic bad channel detection identified, on average, 3.46 (SD = 3.16) noisy and 0.36 (SD = 0.49) flat channels. Following this, the data were bandpass filtered between 0.5 and 125 Hz with a fifth-order Butterworth infinite impulse response (IIR) filter, and notch filters were applied at 50 and 100 Hz to attenuate line noise. The filtered data were then resampled to 250 Hz. Artefactual data segments identified in previous analyses^39,41^ were important and complemented using a window-based outlier detection approach. Magnetometer and gradiometer signals were divided into 2-s epochs, and the standard deviation was computed for each window. A generalised extreme studentized deviate (GESD) test^44^ was used to identify statistical outlier windows, which were marked as bad. This procedure was applied to both raw and differential signals for each sensor type. On average, 15% (SD = ±12) of data were excluded. No bad channels were identified based on their standard deviation profiles. Cardiac and ocular artefacts were removed using signal-space projection (SSP)^45^. One EOG projection and an average of 2 (SD = 0.76) ECG projections were removed per participant. In one case, independent component analysis (ICA) was used instead of SSP due to poor ECG signal quality. To confirm effective artefact attenuation, power spectra of all channels were visually inspected both before and after applying SSP or ICA. MEG recordings from two participants who could not be adequately cleaned were excluded from further processing and analysis.

MEG and MRI data were co-registered using RHINO (Registration of Headshapes Including NOse). Headshape points corresponding to the nose were excluded, and brain surfaces were computed without the nose prior to alignment with anatomical space, due to suboptimal field of view selection in clinical MRIs. Source reconstruction was performed following sensor normalisation^46^ and broadband filtering between 1 and 45 Hz. A forward model was computed, and source estimation was carried out using a linearly constrained minimum variance (LCMV) vector beamformer^46,47^ with principal component analysis (PCA) regularisation set to a rank of 60. Source estimates were projected onto an 8-mm MNI152 standard brain template. The resulting source-level time series were then parcellated using a reduced version^48^ of the Glasser atlas^49,50^. For each parcel, the first principal component of the voxel time courses was extracted to represent the parcel’s activity.

Prior to HMM analyses, symmetrical multivariate leakage correction and sign-flipping were applied. To reduce spatial leakage and eliminate spurious correlations, symmetrical multivariate leakage correction was used^51^, addressing both direct and inherited signal spread as well as so-called “ghost interactions”^52^. Sign-flipping was applied to address the 180-degree ambiguity in dipole orientations by aligning the signs of parcel time courses across participants.

### Preprocessing of STN channel

One contact per subthalamic nucleus (STN) was selected based on the best clinical outcome, assessed 3–6 months post-implantation^40^. Outcomes were determined by a clinician as the most effective therapeutic response to DBS without side effects. The selected contact was re-referenced to the mean signal across all remaining contacts, excluding those identified as bad. Contacts whose time course standard deviation significantly deviated from the other contacts were marked as bad.

### TDE-HMM

A time-delay embedded hidden Markov model (TDE-HMM) was applied to extract dynamic large-scale cortical networks from the MEG recordings. TDE-HMMs provide temporally resolved estimates of network activation, modelling the data as a sequence of transient, recurring brain states that are visited over time. Each state corresponds to a large-scale cortical network defined in a data-driven manner by a unique pattern of multi-region oscillatory activity, i.e., covariations in spectral power and phase synchrony. Contiguous time periods during which a given state is inferred to be active are commonly referred to as visits to that state.

TDE-HMMs represent source-localised and standardised time courses as a sequence of mutually exclusive hidden states. Each state is defined by an observation model that captures distinct auto and cross-covariance patterns across regions. To estimate these patterns, time series samples are time-delay embedded with ±7 samples and reduced to 120 principal components using a PCA before covariance estimation. Importantly, the inferred auto- and cross-covariance patterns capture distinct multiregional spectral power and phase-locking patterns.

The state fitting is typically performed on the whole dataset. However, for small or noisy datasets where reliable state estimation is challenging, it has been proposed to use canonical state definitions derived from external datasets^20^. This approach ensures that well-characterised state descriptions guide the extraction of individual state occurrence probability time courses, based on the assumption that similar states exist across datasets and conditions. Importantly, these predefined states are only used to infer when each network is activated over time in the new dataset. Participant-specific network patterns can still be estimated by pooling data from periods in which each state occurs within the participant’s time series, using a multi-taper approach described below. Given that the PD participants in this dataset were recorded post-surgery with externalised DBS leads, we adopted this strategy.

Running TDE-HMM analyses requires the a priori specification of the number of states that are inferred. Standard model selection metrics often fail to identify a clear optimum number of states for TDE-HMM analyses. Accordingly, it is common practice to choose a relatively low number of states that provides a compact description of the data while demonstrating that key findings are robust across different state numbers^17,20–23,26^. In the main part of this study, we show findings for the HMM inferring eight states. To reassure that the presented findings are similar across variations in the number of states hyperparameter, we repeated all analyses on outputs of HMMs inferring 10 and 12 states.

### State-specific STN-power and STN-cortical coherence

Participant-specific spectral state descriptions were estimated post hoc with a multi-taper pooling across all visits to a state in the respective participant’s time series. More specifically, a multi-taper is applied to the source-reconstructed data, weighted by the a-posteriori state occurrence probabilities inferred with the TDE-HMM^53^. This approach enables the estimation of distinct oscillatory activity patterns within the same cortical region, depending on which large-scale cortical network is active. Time-resolved power and coherence were computed using this multi-taper approach with seven Slepian tapers and a 2-s window, yielding 2 Hz spectral smoothing. Importantly, spectral estimates are calculated across all periods in which a given network occurred, rather than for single visits to a specific network. For visualisation, coherence values were thresholded at the 98th percentile to highlight the strongest connections. For comparisons of time-averaged and state-specific STN-SMA coherence, only ipsilateral STN-SMA coherence values were used and averaged across hemispheres before the calculation of contrasts.

### Overlap between cortical networks and STN beta bursts

To investigate the overlap between STN beta bursts and cortical large-scale networks, STN beta bursts were extracted using the NeuroDSP toolbox^54^. Z-scored time courses from the best clinical contact in each hemisphere were averaged, and bandpass filtered between 13 and 30 Hz, before the amplitude envelope was computed. For each scan, beta bursts were defined as periods where the amplitude envelope exceeded the 75th percentile of that scan’s envelope for at least 100 ms.

Temporal overlap between large-scale cortical network occurrences and STN beta bursts was quantified as the percentage of time both were simultaneously active relative to the respective cortical network’s occurrence time. To assess whether specific networks exhibited significantly greater overlap with STN beta bursts, the overlap with all networks excluding the one of interest was computed for comparison.

### Replication analysis

To assess the generalisability of the findings, key analyses were replicated using an openly available LFP-MEG dataset comprising 20 Parkinson’s disease patients^27^. Preprocessing followed the same pipeline as the main dataset. However, source reconstruction was performed in FieldTrip using individual head and source models provided with the dataset, as structural MRI scans were not available. During preprocessing, three participants were excluded: one due to poor data quality, one due to a missing source model, and one with MEG data only in the medication-off condition. A more detailed description of the demographics and clinical variables of the included sample can be found in Table 1.

In the absence of clinical information about the optimal DBS contact, the STN contact with the highest beta power was selected. Additionally, a 3 Hz high-pass filter was applied prior to TDE-HMM fitting to suppress excessive low-frequency noise (>3 Hz), which otherwise biased the TDE-HMM state fitting. All remaining analyses were conducted identically to the main study.

### Statistics

Within-subject first-level GLMs were used to compare variables between On- and Off-medication states and overlaps between STN bursts and cortical networks. Significance was assessed via maximum t-statistic permutation tests with 1000 permutations, pooling across states to account for multiple comparisons^55^. Power and coherence spectra were compared using cluster-based permutation tests with 1000 permutations. The cluster-forming thresholds were determined as the 0.05-significance threshold of a parametric *t*-test with appropriate degrees of freedom. When these tests were repeated across multiple networks, Bonferroni correction was applied, and only clusters with corrected *p* values below the adjusted threshold were considered significant. Bonferroni correction of the cluster-level *p* values was necessary to account for the increased risk of false positives when comparing multiple networks. The association between STN beta power of both hemispheres and contralateral bradykinesia/rigidity scores was calculated using repeated measures correlation as implemented in Pingouin^56^. PD vs. HC group differences in motor cortical beta power and sensorimotor network fractional occupancy were tested using group-level GLMs with age and sex included as covariate regressors.

### Software

All analyses were performed in Python 3.8. The preprocessing was conducted with the Oxford Software Library for the Analysis of Electrophysiological Data^57,58^ (osl-ephys) (https://github.com/OHBA-analysis/osl-ephys) and MNE^59^ (https://mne.tools/stable/index.html). Spectral and TDE-HMM Analyses were run with OSL-Dynamics (https://github.com/OHBA-analysis/osl-dynamics), burst analysis with NeuroDSP^54^ (https://github.com/neurodsp-tools/neurodsp) and statistical analyses of group and medication contrasts were performed with GLMTOOLs^60^ (https://pypi.org/project/glmtools/). The analysis scripts are publicly available on GitHub (https://github.com/Kohliver/Subcortical-Cortical-Networks-in-PD-).

## Supplementary information

41531_2026_1372_MOESM1_ESM

## Data Availability

The conditions of our ethics approval do not permit public archiving of the data supporting this study. Sharing data requires a formal data-sharing agreement following ethics procedures governing the re-use of sensitive data. Readers seeking access to the data should contact the first author.

## Code availability

All analysis scripts are publicly available on GitHub: https://github.com/Kohliver/Subcortical-Cortical-Networks-in-PD-.
