# Supplementary material for: Varying patterns of association between cortical large-scale networks and subthalamic nucleus activity in Parkinson’s disease
Source: NPJ Parkinsons Dis. 2026 May 2;12:106. doi: 10.1038/s41531-026-01372-1 (PMC13135510; doi:10.1038/s41531-026-01372-1)
Supplement: Supplementary file 1 — 41531_2026_1372_MOESM1_ESM [file 41531_2026_1372_MOESM1_ESM.pdf]

Supplementary Information: Varying patterns of association between cortical large-scale networks and subthalamic nucleus activity in Parkinson's Disease

Oliver Kohl[1], Chetan Gohil[2], Matthias Sure[1], Alfons Schnitzler[1,3], Esther Florin[1]

Table of Contents

Supplementary Figure 1 – Motor cortical beta power and sensorimotor network fractional occupancy are reduced in PD compared to HCs 2

Supplementary Figure 2 - State-specific absolute imaginary coherence between STN and SMA . 4

Supplementary Figure 3 – State-specific STN Beta Power 5

Supplementary Figure 4 & 5 - Robustness of Effects 6

Supplementary Figure 6 – Replication HMM Overview 11

Supplementary Figure 7 – State-specific STN-premotor coherence 13

Supplementary Information 1 – Discussion of motor cortical beta power reduction in PD compared to HCs 14

Supplementary Figure 1 – Motor cortical beta power and sensorimotor network fractional occupancy are reduced in PD compared to HCs

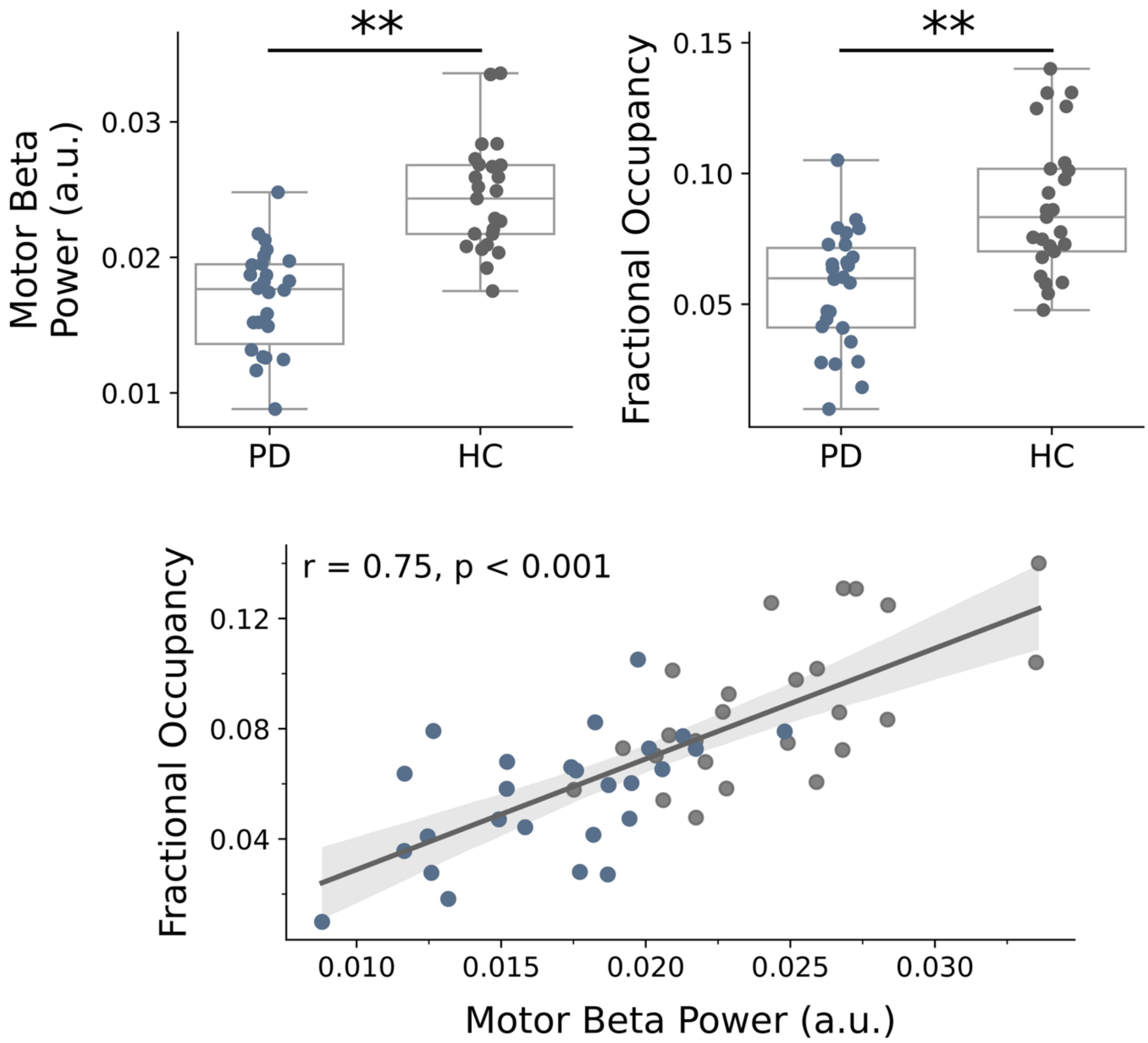

Supplementary Figure 1. Reductions in sensorimotor network occurrence probability underpin reductions in motor cortical beta power in PD.

Top left: Motor cortical 13 to 30-Hz power of people in PD (blue) is significantly reduced to healthy controls (grey) ($t(47) = 6.78$, $p < .001$). Significance was calculated with GLMs including age and sex as confounds. **Top right:** Sensorimotor network fractional occupancy of people in PD (blue) is significantly reduced compared to healthy controls (grey) ($t(47) = 4.48$, $p < .001$). Significance was calculated with GLMs contrasting fractional occupancy of all 8 states between the two groups while including age and sex as confounds and maximum t-statistic pooling to account for multiple comparisons across states. **Bottom:** Sensorimotor network fractional occupancy and beta power are positively associated ($r(49) = .75$, $p <$

.001). Each dot corresponds to fractional occupancy and beta power of a single participant. Associations were calculated with a Pearson correlation.

Asterisks denote statistical significance: ** $p < 0.01$; * $p < 0.05$

Supplementary Figure 2 - State-specific absolute imaginary coherence between STN and SMA

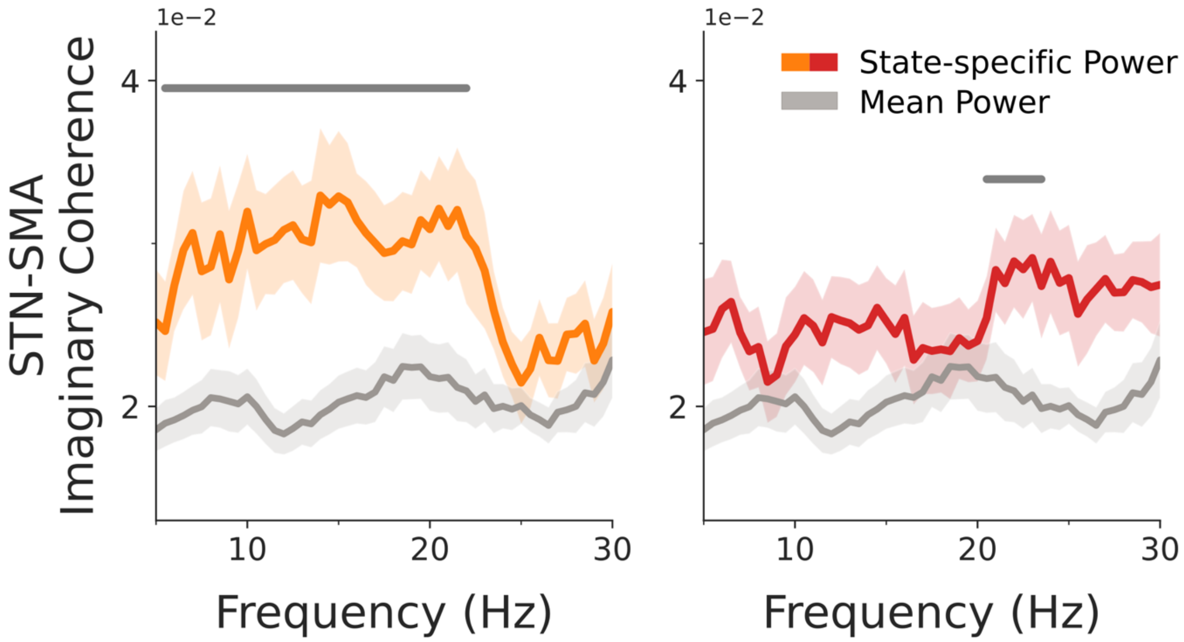

Supplementary Figure 2. State-specific absolute imaginary coherence between STN and SMA demonstrates similar connectivity patterns as state-specific coherence. Within-participant GLMs combined with cluster-based permutation testing revealed that absolute imaginary coherence between STN and SMA of the widespread activation network (orange) is significantly larger in the 5.5 to 23-Hz range than the mean absolute imaginary coherence across all states (peak frequency = 12-Hz; mean $t(24) = 3.08$, $p < .001$). Similarly, absolute imaginary STN-SMA coherence was significantly increased in the 20.5 to 24.5-Hz range during sensorimotor network occurrences (peak frequency = 23-Hz; mean $t(24) = 2.53$, $p = .023$). Grey bars indicate clusters with p-values below the Bonferroni-corrected threshold for 2 state comparisons ($p < 0.025$).

Supplementary Figure 3 – State-specific STN Beta Power

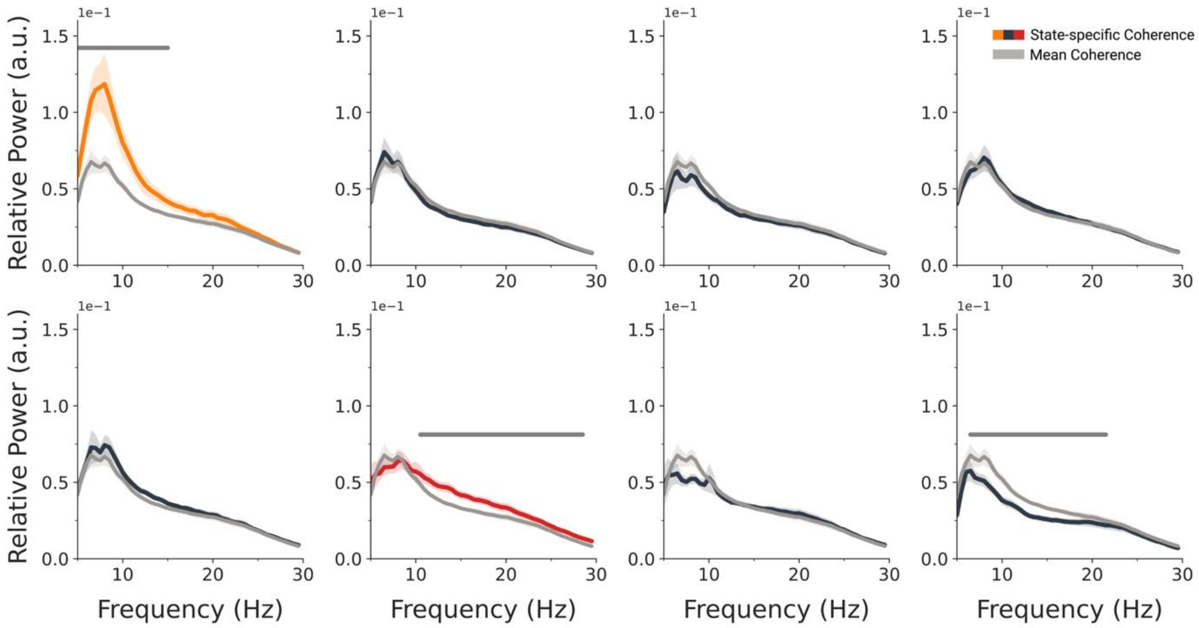

Supplementary Figure 3. STN power spectra during cortical large-scale network occurrences. State-specific STN power spectra for the widespread activation network (orange), the sensorimotor network (red), and all other networks (black) are shown alongside the time-averaged STN power spectrum across all states (grey). Cluster-based permutation testing on within-participant GLMs revealed significantly increased STN power during visits to the widespread activation network (5 to 16.5-Hz; peak = 6.5 Hz; mean t(24) = 2.91, $p <$ .001) and the sensorimotor network (9.5 to 23-Hz; peak = 14 Hz; mean $t(24) = 3.41$, $p =$ .001). In contrast, significant 6.5 to 29.5-Hz power decreases were observed for periods in which State 8 was visited (peak = 6 Hz; mean $t(24) = -4.64$, $p < .001$). Grey bars indicate clusters surviving Bonferroni correction for eight state comparisons ($p < 0.00625$).

Supplementary Figure 4 & 5 - Robustness of Effects

Running TDE-HMM analyses requires the a-priori specification of the number of states to be inferred. Since different numbers of states yield similar but slightly different network descriptions, we repeated all analyses on TDE-HMMs inferring 10 and 12 States to demonstrate that the reported findings are not limited to the choice of 8 states but are also present when selecting other numbers of states.

Accordingly, we fitted TDE-HMMs with 10 and 12 States. To identify the states corresponding to the widespread activation and sensorimotor networks observed in the 8-state model, we computed spatial correlations between wideband power maps of these networks and all states from the 10- and 12-state TDE-HMMs. This analysis revealed that, in both the 10- and 12-state HMM fits, one state was highly correlated across all participants with the sensorimotor network (10-state HMM: State 8; 12-state HMM: State 9), while another state was highly correlated with the widespread activation network (10-state HMM: State 1; 12-state HMM: State 1; Supplementary Figure 4). These networks were selected for subsequent analyses to assess the robustness of the HMM findings.

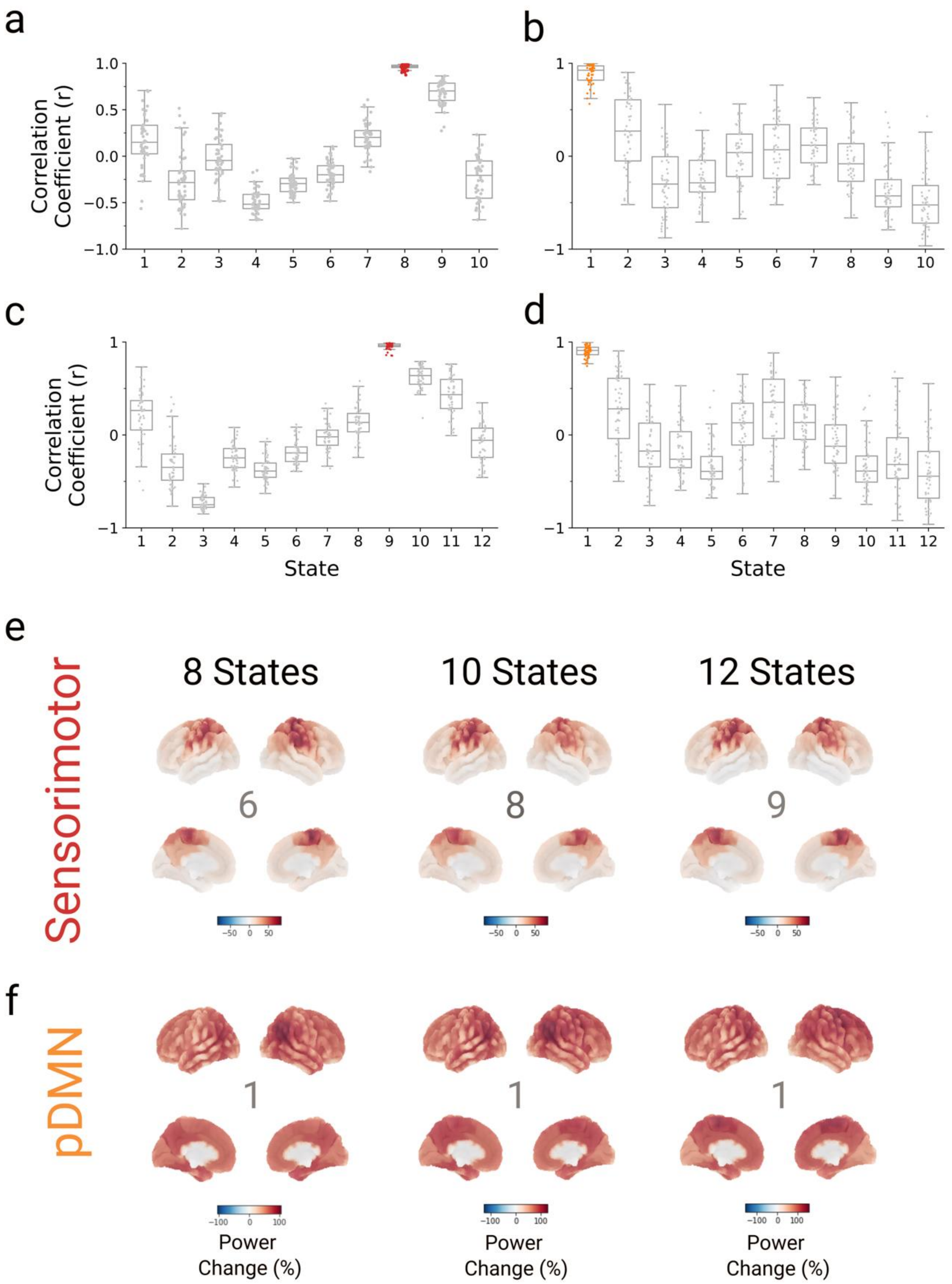

Supplementary Figure 4. Matching of states across TDE-HMMs with different numbers of states. Spatial correlations between wideband power maps of the sensorimotor network obtained from a TDE-HMM with 8 states and those from TDE-HMMs with 10 (**a**) and 12 states (**b**). Spatial correlations between the posterior default mode network (pDMN) of the 8-state HMM and all states identified by the 10-state (**c**) and 12-state (**d**) TDE-HMMs. Each dot represents the Pearson correlation coefficient for a single participant. Below, wideband power

maps projected onto the cortical surface are shown for the matched sensorimotor networks (**e**) and the pDMN (**f**). The state number corresponding to each TDE-HMM fit is indicated at the centre of each map. Power maps represent the percentage change in wideband power during occurrences of each state relative to the mean wideband power across all states.

Repeating analysis on state-specific STN cortical-coherence for 10-State and 12-State HMMs also revealed significant increases in STN-SMA coherence for both the widespread activation and sensorimotor network, albeit with differences spreading further across the majority of frequency bands. In the 10-State HMM an significant increases in STN-SMA coherence in the 5 to 30Hz range were observed for the widespread activation network and significant increases in the 5 to 18-Hz (peak frequency = 9.5-Hz; mean $t(24) = 3.40$, $p < .001$) and 16.5 to 27.5 (peak frequency = 26.5-Hz; mean $t(23) = 3.51$, $p = .002$) range for the sensorimotor network. For the 12 State HMM, significant increases in 5 to 30Hz STN-SMA coherence were observed both during visits to the widespread activation (peak frequency = 5.5-Hz; mean $t(24) = 5.55$, $p < .001$) and sensorimotor network (peak frequency = 9.5-Hz; mean $t(24) = 3.84$, $p < .001$).

Similar to the 8 State HMM analyses of state-specific STN power spectra revealed for the 10-State HMM, significant increases in the low frequency range (6.5 to 7-Hz; peak frequency = 6.5-Hz; mean $t(24) = 1.87$, $p < .001$) for the widespread activation network and significant increases in the 10 to 23-Hz range (peak frequency = 13.5-Hz; mean $t(24) = 2.63$, $p = .002$). Low frequency increases during visits to the widespread activation network, identified from the 12-State HMM, failed to reach significance, whereas significant increases in the 10 to 22.5-Hz range (peak frequency = 18-Hz; mean t(24) = 3.84, p = .002) were replicated.

In line with findings in the 8-State HMM, widespread activation network occurrences did not significantly overlap with STN-beta bursts in the 10-State ($t(24) = 2.17$, $p = .068$) and 12-State HMMs ($t(24) = 1.97$, $p = .106$), whereas sensorimotor network occurrences did (10-State HMM: $t(24) = 3.2$, $p = .004$; 12-State HMM: $t(24) = 2.94$, $p = .008$)

Repetition of contrasts between state-specific beta power in the medication on and off conditions revealed that beta power was significantly changed only during visits to networks that do not exhibit increased levels of STN-SMA coherence, for both State-10 HMMs and 12-State HMMs. In the 10-State HMM significant decreases in state-specific beta power upon medication intake were observed for State 3 ($t(24)$=3.80, $p = .005$), State 4 ($t(24)$=3.79, $p = .005$), State 9 ($t(24)$=3.83, $p = .005$), and State 10 ($t(24)$=3.64, $p = .008$). In the 12 State-HMM state-specific STN beta power was reduced for State 4($t(24)$=4.07, $p = .002$), State 5

(*t*(24)=3.47, *p* = .011), State 10 (*t*(24)=3.27, *p* = .019), State 11 (*t*(24)=4.35, *p* = .001), State 12 (*t*(24)=3.75, *p* = .006). See Supplementary Figure 5 for an overview of all these findings.

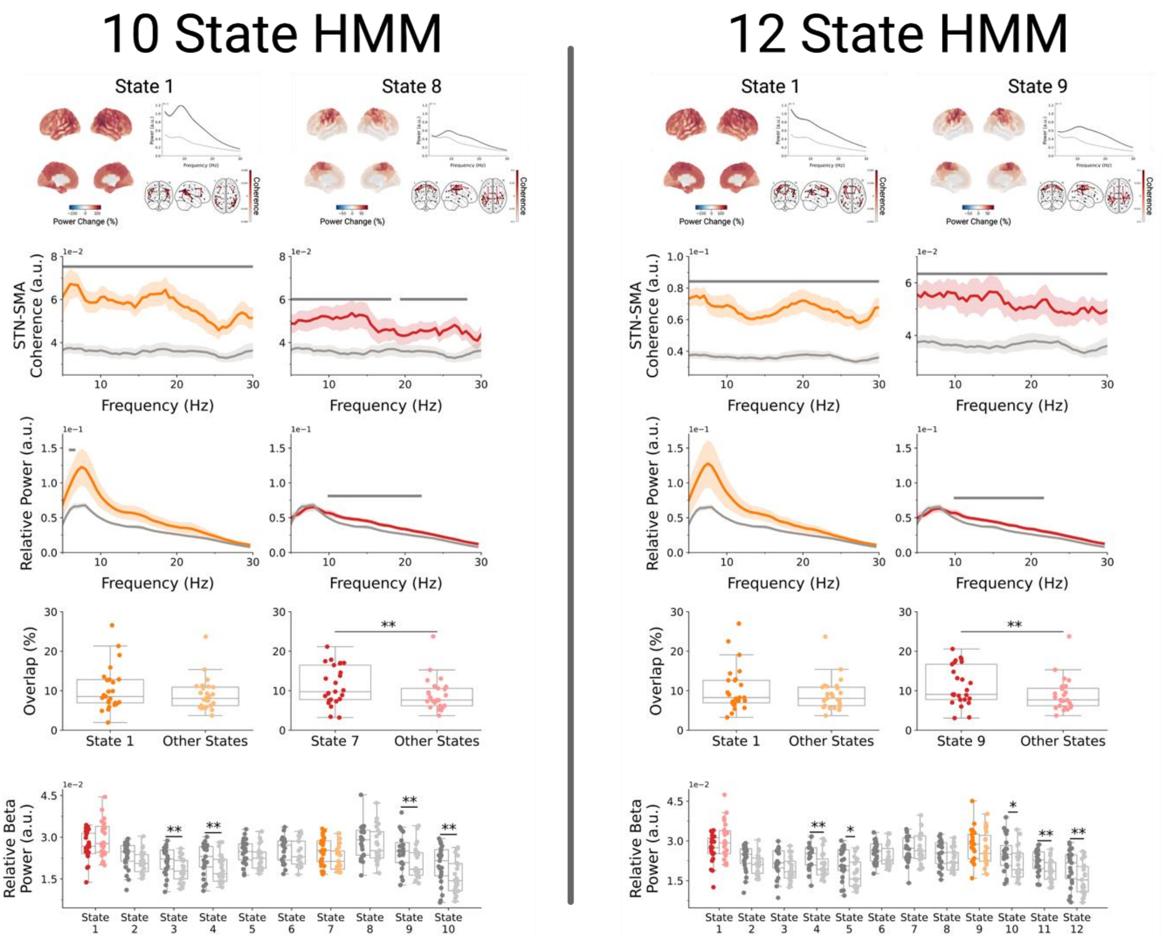

Supplementary Figure 5. 10-State and 12-State HMMs show similar patterns of change in spectral markers during widespread activation network and sensorimotor network visits. Findings for the 10-State HMM are presented on the left, and findings for the 12-State HMM on the right side. **Top row**: State descriptions of the widespread activation and sensorimotor network inferred with respective HMM. **Second row**: STN-SMA coherence for both the widespread activation network (orange) and sensorimotor network (red) is contrasted against time-average STN-SMA coherence (grey). **Third row**: State-specific STN power spectra for both the widespread activation network (orange) and sensorimotor network (red) are contrasted against the time-averaged STN power spectrum (grey). **Fourth row**: Overlap between STN beta burst and occurrences of the widespread activation network (orange) or sensorimotor network (red) are contrasted against overlaps between STN beta bursts and all states excluding the respective state (shaded orange and shaded red). Each dot represents the overlap of a single participant. **Bottom row**: Contrast of state-specific STN beta power

between medication off- (strong colours) and off-condition (shaded colours). The widespread activation network (orange) and sensorimotor network (red) are highlighted with colours. Grey bars indicate clusters surviving Bonferroni correction for eight state comparisons ($p < 0.00625$).

Asterisks denote statistical significance: ** $p < 0.01$; * $p < 0.05$

Supplementary Figure 6 – Replication HMM Overview

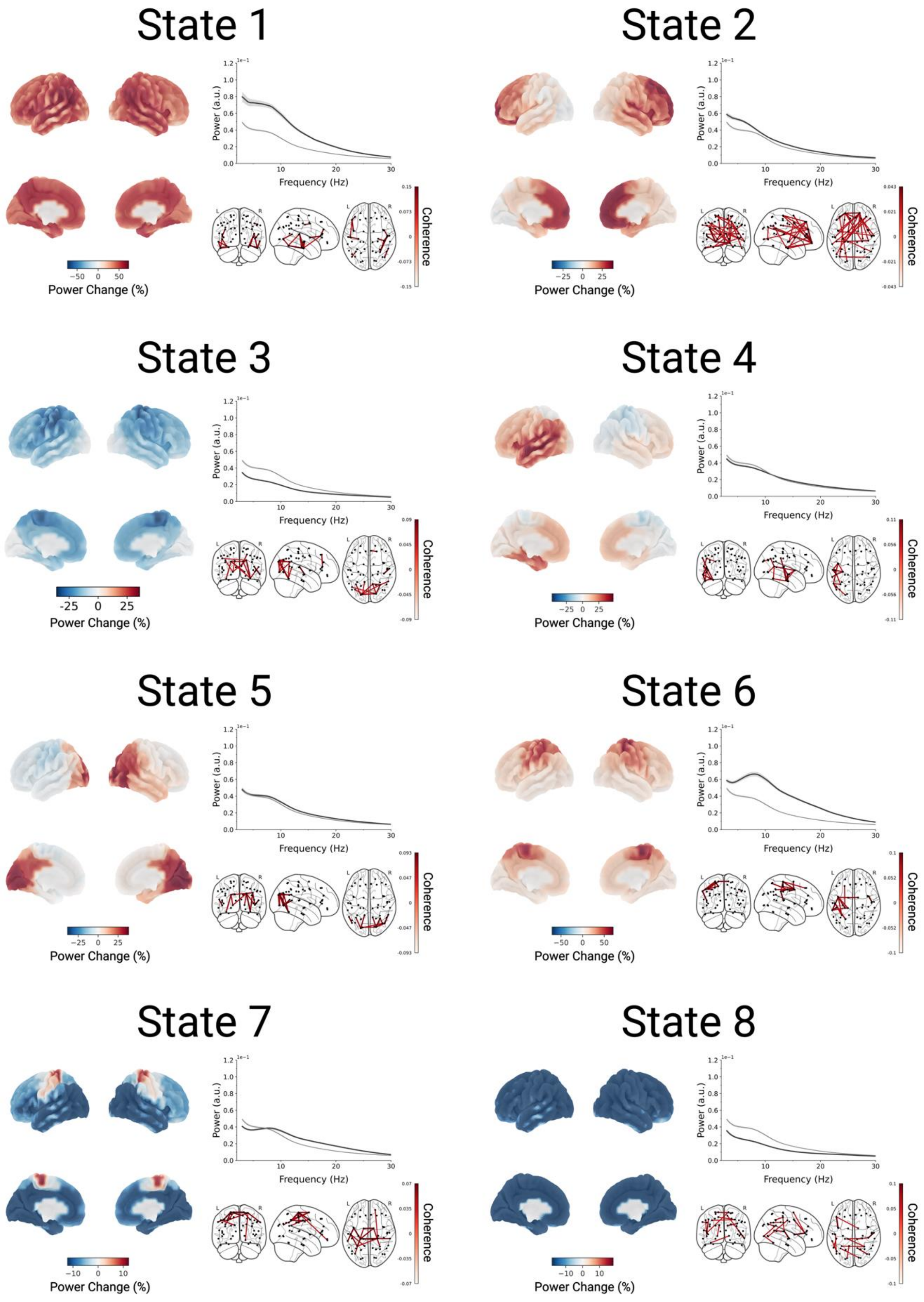

Supplementary Figure 6 Overview of dynamic large-scale cortical networks inferred from the replication dataset[1] using the TDE-HMM. Each state is presented in three panels, showing the average across scans from all participants in both the medication-off and -on conditions. **Left panel:** Deviations in 3 to 30-Hz power from the time-averaged power (calculated across all states) are projected onto the cortical surface. **Top right panel:** The state-specific motor cortical power spectrum (black) is displayed alongside the time-averaged power spectrum across all states (grey). **Bottom right panel:** Coherence networks in the 2 to 30-Hz range, thresholded at the 98th percentile, to highlight the most prominent functional connections. State 1 corresponds to the widespread activation network, and State 6 to the sensorimotor network.

Supplementary Figure 7 – State-specific STN-premotor coherence

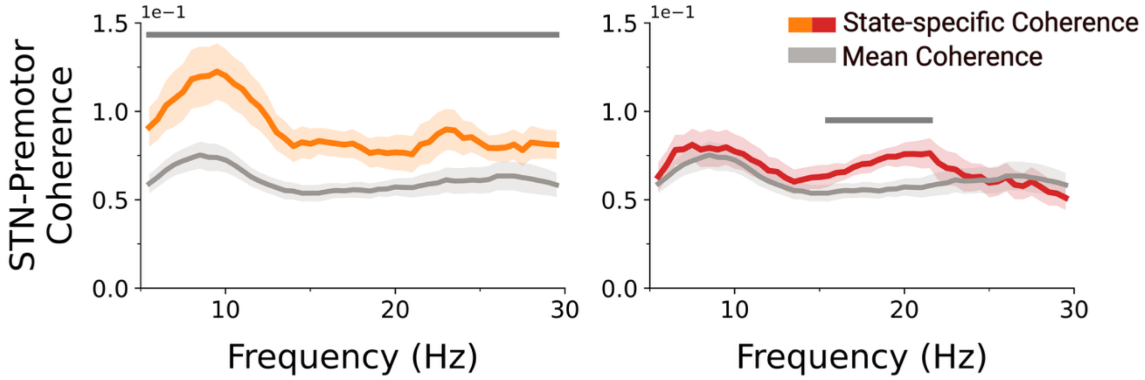

Supplementary Figure 7 State-specific STN-Premotor coherence (dark orange, dark red) is shown alongside the time-averaged coherence across all states (grey). Within-participant GLMs, combined with cluster-based permutation testing, revealed significantly increased STN-Premotor coherence during visits to the widespread activation network (State 1; orange; 5.5-29.5 Hz; peak = 16.5 Hz; mean $t(16) = 3.49$, $p < 0.001$) and the sensorimotor network (State 6; red; 15.5-21.5 Hz; peak = 19 Hz; mean $t(16) = 3.63$, $p = 0.002$).
Grey bars indicate clusters with $p < 0.00625$ (Bonferroni-corrected threshold for 8 states).

Supplementary Information 1 – Discussion of motor cortical beta power reduction in PD compared to HCs

In the main part of the manuscript, we observed that STN beta bursts significantly co-occurred with sensorimotor network occurrences, suggesting a close link. Simultaneously, we observed a reduction in sensorimotor network occurrences in individuals with PD compared to HCs, while STN beta power was decreased following dopaminergic medication. These findings point to a nuanced relationship between subcortical beta activity and cortical network dynamics in PD that goes beyond the notion that cortical sensorimotor network activity simply reflects beta hypersynchronisation throughout the basal ganglia-thalamo-cortical loop, blocking information processing capacity.[2,3]

Although STN beta bursts and sensorimotor network activity (or motor cortical beta bursts[2,4]) overlap in time, they do not exhibit a perfect one-to-one correspondence. In our data, STN beta bursts co-occurred with < 25% of sensorimotor network occurrences, consistent with previous reports of < 30% overlap for motor cortical bursts.[4],[2] This partial overlap suggests that additional processes contribute to beta activity in each region: STN beta activity also reflects basal ganglia circuit activity,[4–8] and motor cortical beta activity also captures widespread oscillatory slowing in PD.[9–17]

Our findings may be particularly sensitive to such widespread changes because sensorimotor parcel time courses were extracted as the first principal component across all voxels within each parcel. Given that these parcels span relatively large cortical regions, this approach likely emphasises widespread alterations such as oscillatory slowing over more localised dynamics. The potential bias of this common practice could also help explain discrepancies between our MEG results and ECoG studies reporting unchanged[18] or increased[19,20] motor cortical beta power in PD compared to people with primary dystonia or essential tremor. The higher spatial resolution of ECoG may enable more precise localisation of basal ganglia-

thalamo-cortical loop activity while being less sensitive to widespread cortical changes.

In sum, these findings highlight the sensorimotor network as a promising non-invasive marker for PD, providing temporally resolved access to subthalamic activity that is otherwise difficult to capture without invasive recordings. At the same time, it is likely sensitive to broader cortical phenomena, highlighting the need for careful interpretation and for approaches that can further disentangle disease-specific basal ganglia-thalamo-cortical dynamics from more widespread cortical changes.

STROBE Statement—Checklist of items that should be included in reports of ***cross-sectional studies***

	Item No	Recommendation	Page
**Title and abstract**	1	(*a*) Indicate the study's design with a commonly used term in the title or the abstract	Page 2<br>28 -31
		(*b*) Provide in the abstract an informative and balanced summary of what was done and what was found	Page 2<br>28 -31
**Introduction**			
Background/rationale	2	Explain the scientific background and rationale for the investigation being reported	Page 2-3<br>41-64
Objectives	3	State specific objectives, including any prespecified hypotheses	Page 3-4<br>65-78
**Methods**			
Study design	4	Present key elements of study design early in the paper	Page 4<br>80-84
Setting	5	Describe the setting, locations, and relevant dates, including periods of recruitment, exposure, follow-up, and data collection	Page 14<br>331-337
Participants	6	(*a*) Give the eligibility criteria, and the sources and methods of selection of participants	Page 14<br>331-337
Variables	7	Clearly define all outcomes, exposures, predictors, potential confounders, and effect modifiers. Give diagnostic criteria, if applicable	
Data sources/ measurement	8*	For each variable of interest, give sources of data and details of methods of assessment (measurement). Describe comparability of assessment methods if there is more than one group	Page 14<br>339-353
Bias	9	Describe any efforts to address potential sources of bias	Page 17-18<br>418-424<br>Page 19<br>449-459
Study size	10	Explain how the study size was arrived at	Page 16<br>372-373
Quantitative variables	11	Explain how quantitative variables were handled in the analyses. If applicable, describe which groupings were chosen and why	Page 19-20<br>461-473
Statistical methods	12	(*a*) Describe all statistical methods, including those used to control for confounding	Page 19-20<br>461-473
		(*b*) Describe any methods used to examine subgroups and interactions	/
		(*c*) Explain how missing data were addressed	Page 16<br>372-373
		(*d*) If applicable, describe analytical methods taking account of sampling strategy	/
		(*e*) Describe any sensitivity analyses	/
**Results**			
Participants	13*	(a) Report numbers of individuals at each stage of study—eg numbers potentially eligible, examined for eligibility, confirmed eligible, included in the study, completing follow-up, and analysed	Page 4<br>80-84<br>Page 16<br>372-373
		(b) Give reasons for non-participation at each stage	Page 16

			372-373
		(c) Consider use of a flow diagram	/
Descriptive data	14*	(a) Give characteristics of study participants (eg demographic, clinical, social) and information on exposures and potential confounders	Page 34-35 717-721
		(b) Indicate number of participants with missing data for each variable of interest	Page 34-35 717-721
Outcome data	15*	Report numbers of outcome events or summary measures	Page 4-9 86-223
Main results	16	(*a*) Give unadjusted estimates and, if applicable, confounder-adjusted estimates and their precision (eg, 95% confidence interval). Make clear which confounders were adjusted for and why they were included	Page 4-9 86-223
		(*b*) Report category boundaries when continuous variables were categorized	/
		(*c*) If relevant, consider translating estimates of relative risk into absolute risk for a meaningful time period	/
Other analyses	17	Report other analyses done—eg analyses of subgroups and interactions, and sensitivity analyses	/
**Discussion**			
Key results	18	Summarise key results with reference to study objectives	Page 10 226-238
Limitations	19	Discuss limitations of the study, taking into account sources of potential bias or imprecision. Discuss both direction and magnitude of any potential bias	Page 12-13 289-322
Interpretation	20	Give a cautious overall interpretation of results considering objectives, limitations, multiplicity of analyses, results from similar studies, and other relevant evidence	Page 10 226-238
Generalisability	21	Discuss the generalisability (external validity) of the study results	Page 11 263-272 Page 13 302-322
**Other information**			
Funding	22	Give the source of funding and the role of the funders for the present study and, if applicable, for the original study on which the present article is based	Page 21 491-493

*Give information separately for exposed and unexposed groups.

Note: An Explanation and Elaboration article discusses each checklist item and gives methodological background and published examples of transparent reporting. The STROBE checklist is best used in conjunction with this article (freely available on the Web sites of PLoS Medicine at http://www.plosmedicine.org/, Annals of Internal Medicine at http://www.annals.org/, and Epidemiology at http://www.epidem.com/). Information on the STROBE Initiative is available at www.strobe-statement.org.
